# The effect of a multi-component camp-based weight-loss program on children’s motor skills and physical fitness: a randomized controlled trial

**DOI:** 10.1186/s12887-016-0627-5

**Published:** 2016-07-15

**Authors:** Kristian Traberg Larsen, Tao Huang, Lisbeth Runge Larsen, Line Grønholt Olesen, Lars Bo Andersen, Niels Christian Møller

**Affiliations:** Centre of Research in Childhood Health (RICH), Department of Sports Science and Clinical Biomechanics, University of Southern Denmark, Odense, Denmark; Department of Physical Education, Shanghai Jiao Tong University, Shanghai, China; Research & Innovation Center for Human Movement & Learning (FIIBL), Department of Sports Science and Clinical Biomechanics, University College Lillebaelt, Odense, Denmark; Institute of Regional Health Research, University of Southern Denmark, Odense, Denmark; Department of Sports Medicine, Norwegian School of Sport Sciences, Oslo, Norway; Faculty of Teacher Education and Sport, Sogn og Fjordane University College, Sogndal, Norway

**Keywords:** Children, Overweight, Obesity, Motor skills, Physical fitness, Multi-component intervention, Weight loss, RCT

## Abstract

**Background:**

Many weight-loss programs in children are performed without specific foci on training both physical fitness and motor skills. The aim of this study was to describe the effect of a one-year weight-loss program on children’s motor skills and physical fitness.

**Methods:**

Participants included 115 overweight fifth-grade children (12.0 years) randomized into either a Day-Camp Intervention Arm (DCIA), with a subsequent family-based support program or a low-intense Standard Intervention Arm (SIA). Physical fitness was assessed by vertical jump, hand grip strength, and a progressive cardio-respiratory fitness test. Motor skills were assessed by the Movement Assessment Battery for Children – second edition (M-ABC-2), age band 3.

**Results:**

Loss to follow-up after 52 weeks was 19 % and 32 % in the DCIA and SIA, respectively. Balance skills were improved post-camp, but not after 52 weeks in children from the DCIA compared to the SIA. Contrary to the expected, children from the SIA improved aiming and catching skills relative to the DCIA children. Overall z-scores of the physical fitness components and cardio-respiratory fitness improved more in children from the DCIA compared to children from the SIA.

**Conclusion:**

In conclusion, the day-camp intervention led to improvements in physical fitness but not in motor skills compared to the standard intervention. Including both motor skills and physical fitness could advantageously be considered in future immersive intervention programmes.

**Trial registration:**

Clinicaltrials NCT01574352, March 26, 2012 (retrospectively registered).

## Background

Being overweight as a child or adolescent also increases the risk of adult morbidity and mortality [1, 2]. Consequently, there is a need for effective and sustainable approaches for weight-loss interventions in children and adolescents. The most effective treatment of obesity is suggested to be multi-disciplinary, thus, consisting of several different components [3]. In this context, a promising approach is immersive treatment strategies in which overweight children are removed from their obesogenic environments and placed in therapeutic and educational surroundings [4]. Continued participation in physical activity (PA) have shown to be an important element in achieving success, both in terms of weight loss and long-term sustainability [3]. The majority of existing long-term evaluations of weight-loss programs with PA as one of the essential foci have documented a general relapse of the attained weight loss, typically after one to two years [3]. To avoid this, it seems critical to incorporate elements, such as continued participation in PA, in the intervention program in order to support the sustainability of the participants’ achieved weight loss.

Both motor skills (MS) and physical fitness (PF) affects the level of physical activity in children [5–10]. A higher level of MS has been linked to an increased self-reported PA level among school children [7], and an inverse relationship between MS and being overweight also seems to be present in children [5, 8]. Evidence also suggests that failure to attain a basic level of MS may contribute to a decline in PA during adolescence [9, 10]. Moreover, PF contains elements of health-related fitness (e.g. cardiovascular fitness and muscular strength) and performance-related fitness (e.g. power and balance) [6]. Consequently, if overweight children’s performance in MS and PF can be enforced during a weight-loss intervention program, the chances of sustained weight loss through increased engagement in PA would increase [11]. Numerous immersive weight-loss interventions conducted in children have PA as a key component, but to our knowledge only one previous study has focused on improving both MS and PF [12]. In intervention programs containing various and intensive daily PA components, it could be assumed that the increased amount of PA per se would improve MS and PF [11, 13]. Taking into account the reduced level of MS and PF observed in overweight children [5], even exercises of a low motor difficulty could potentially lead to improved MS and PF. However, it remains unknown whether MS and PF can be improved as a result of immersive PA-based weight-loss interventions in children without a specific focus on promoting MS and PF. Therefore, the aim of the present study is to determine how an immersive day-camp intervention programme, with the primary focus of weight loss, is influencing the development of MS and PF in children when compared to a low-intense standard intervention programme.

## Methods

### Study design

The Odense Overweight Intervention Study (OOIS) is a randomized controlled trial designed to compare the effect of a one year multi-component day-camp weight-loss intervention for children with a short-lasting low-intense standard programme. Reporting adheres to the CONSORT guidelines. A study protocol with a more detailed description of methods, intervention components, and analysis strategies has earlier been published [14]. The study protocol was approved by The Regional Scientific Ethical Committee for Southern Denmark (Approval number: S-20120015), registered with ClinicalTrial.gov (Registration number: NCT01574352) before initializing the trial.

### Participants

Fifth-grade children from two consecutive school years in the municipality of Odense, Denmark, were examined for overweight by school nurses. Children with an age and sex specific body mass index (BMI) status corresponding to >25 for adults, were subsequently invited to participate in the trial. Exclusion criteria were: 1) the child was participating in other overweight programs; 2) the child was not attending regular school classes due to behavioural issues; 3) the child had a known clinically diagnosed endogenous cause of overweight; 4) the child had a motor-control handicap which hindered normal participation in PA; or 5) the child had known violent behaviour. Families were invited to a meeting for detailed information about the project. If the children’s parents or legal guardians agreed to participate, these gave written consent before enrolling the children to the trial. Sex-stratified concealed block randomization with a ratio of 1:1 ensured balance between intervention arms.

### Study interventions

#### Day-Camp Intervention Arm (DCIA)

The day camp took place in Odense, Denmark, from the middle of May to the end of June, in 2012 and 2013. The camp lasted for six consecutive weeks, seven days a week, from 7 a.m. until 8.30 p.m. Outside these hours the children stayed at home with their families. Each day of the day-camp, children were engaged in PA classes consisting of minimum three hours of structured exercise with a focus on physical activity enjoyment and motivation (e.g. dancing, team building, and alternative ball-games), one hour of health classes (focused on knowledge, theory, and behaviour change), and one hour of homework assignment (as the intervention took place during school weeks). The camp instructors were giving overall guidelines with the purpose of introducing the children to a large variety of fun-based games and exercises in order to strengthen their confidence in the sporting environment. On this basis, camp instructors were responsible of creating the specific content of the classes. No specific motor skill training was introduced. Healthy food was prepared by trained kitchen staff [15] and the meals were supervised by the camp instructors. No diet restrictions were enforced.

After six weeks of day-camp intervention, a family-based intervention during the subsequent 46 weeks was initiated with the purpose of supporting the families in adopting the lifestyles attained during the day-camp intervention. Parents received written and oral health information e.g. about how to increase habitual PA and prepare healthy food. The responsibility of health behaviour at this stage rested entirely on the parents and children. No specific PA levels were required. Challenges emerging after the day-camp were addressed during four joint meetings with special trained school nurses and instructors from the day-camp. After approximately five months, an activity day was arranged for the children.

#### Standard Intervention Arm (SIA)

The standard intervention programme was designed as a minimum intervention as required by the local ethical committee. It consisted of a single weekly exercise session (two hours duration) for six weeks, as well as one health and lifestyle educational session for the parents, delivered by a dietician and a PA specialist. The standard intervention ran simultaneously with the day-camp and ended after six weeks.

### Data collection

Data was collected at the University of Southern Denmark, Odense, on three separate occasions; at baseline, six weeks follow-up (post day-camp), and at 52 weeks follow-up. As children were going through a significant number of measurements during a test day, the sequence of the tests were planned to be similar for each child on each of their three test days. Test personnel were not aware of children’s allocation.

#### Anthropometrics

Body height was assessed on a wall mounted stadiometer without footwear. Body weight was assessed on a Soehnle Professional Medical electronic scale in underwear. Sexual maturity was assessed according to Tanner’s five pubertal stages by self-evaluation, as described by Malina et al. [16]. Self-assessment of Tanner stages has earlier shown relatively sound agreements with objective assessments [17, 18].

#### Motor skills

Motor skills were assessed with the Movement Assessment Battery for Children - Second Edition (M-ABC-2), age band three (11 to 16 year-olds) [19]. The M-ABC-2 is designed to screen for motor impairment using two components, the Performance Test and the Checklist. For practical reasons, only the product-oriented Performance Test was used in the present study. The test was composed of a series of eight fine and gross motor tasks (items) grouped into three subscales: Manual Dexterity (M_Hand_) composed by 1) turning pegs, 2) triangle with nuts and bolts, and 3) drawing along a visual trail; Aiming and Catching skills (M_Ball_) composed by 4) catching with one hand and 5) throwing ball at wall mounted target; and Balance skills (M_Balance_) composed by 6) two-board balance, 7) walking toe-to-heel backwards, and 8) zig-zag hopping on one leg. Using the M-ABC-2 scoring manual [19], the scores from the tests yielded raw scores and corresponding standard scores from each subscale For study outcomes the standard scores were used, as they represented the weighted performance for each sub-scale. Additionally, a standard score from the overall motor skills (M_Overall_) was extracted from the test. The M-ABC-2 test has previously shown high validity, reliability, and responsiveness to change over time [20, 21].

#### Physical fitness

Handgrip strength (F_Strength_) was assessed using a Smedley Dynamometer. The best of three attempts using the dominating hand were registered. F_Strength_ has been shown to correlate highly with upper body strength in children with high validity [22, 23]. The highest vertical jump height (F_Jump_) was assessed during a counter movement jump. Measuring tape was fixed on the front to a belt and through a moderate resistance on the floor, thus registering the peak height of the jump. The child was allowed to swing their arms during the take-off. If the child improved during the three attempts, he/she would be given another attempt until no further improvements were registered. F_Jump_ correlates well with lower body strength and also constitutes an element of coordination [22, 23]. The maximum oxygen uptake (VO_2peak_) was assessed using a progressive resistance increasing cycle ergometer protocol (Monark Ergomedic 839e) until total exhaustion with indirect calorimetry (AMIS 2001, Innovision) and a Polar RS800CX heart rate monitor. Completion was approved at a stable respiratory exchange rate >1.08 as earlier suggested for children at the age of 11 to 13 years [24]. A previous study has compared the AMIS 2001 to Douglas bag and showed a coefficient of variation of 1.9 % with respect to oxygen uptake [25].

#### Demographics

Information of parental income and ethnicity was collected in a questionnaire at baseline based on the questionnaire used in the Northern Ireland Childhood Coronary Prevention Study [26].

### Study outcomes

Mean standard scores from each subscale and M_overall_ from the M-ABC-2 were used to outline the development of motor skills during the trial. Cardiorespiratory fitness (F_Cardio_) was calculated as mL O_2_ (VO_2peak_)/(min · kg body weight). Physical fitness was reported separately as F_Strength_ (kg), F_Jump_ (cm), and F_Cardio_ and as a sum of z-scores from the three variables, overall physical fitness (F_Overall_). BMI was calculated as body weight (kg) divided by square of body height (m^2^). Parental socio-economic status, derived from self-reported questionnaires, was based on the mother’s highest education level and subsequently dichotomized into high/low according to the International Standard Classification of Occupations from 2008 [27]. Ethnicity, derived from self-reported questionnaires, was dichotomized into Danish/Non-Danish origin.

### Statistical analyses

To describe baseline data, frequencies, means with standard deviations, and medians with inter-quartile ranges were presented. Linear mixed-effects modelling for repeated measures was applied to determine differences between intervention arms in development of standard scores for M_Overall_ and the underlying subscales. Similar mixed-effects models were applied to determine the group differences in development of F_Overall_ and the underlying sub-components. Maximum likelihood estimation was used for all models [28]. Akaike information criterion and Bayesian information criterion determined whether random intercepts or random slope models were preferred. Unstructured covariance matrix was applied when a random slope model was used. The normality of first level residuals, random intercepts and slopes, as well as homoscedasticity, were investigated in all models. For all statistical analysis, Stata version 12.1 SE (StataCorp LP, College Station, TX, USA) was used.

## Results

### Baseline characteristics and trial flow

**Fig. 1:**
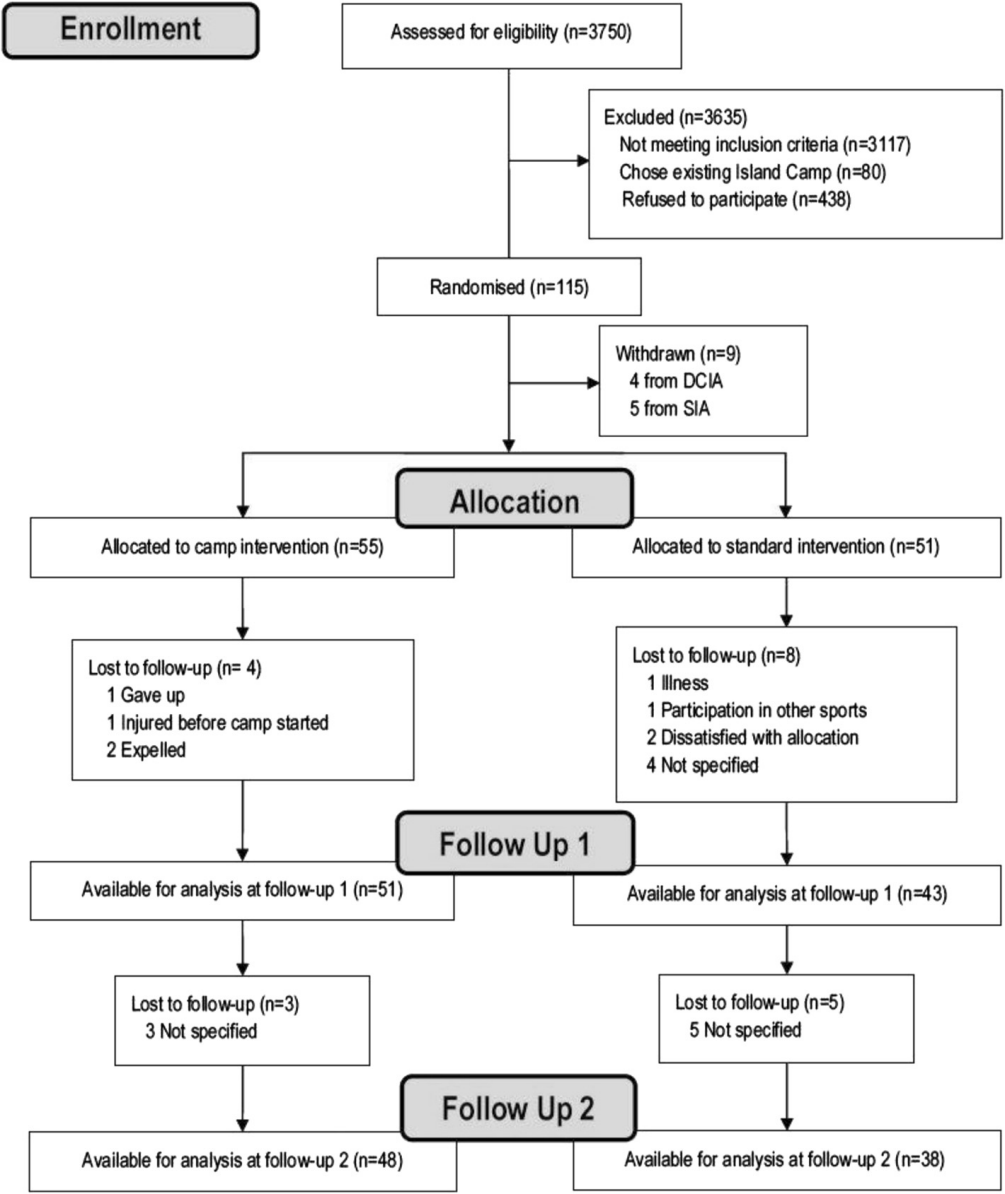
Flow of participants

Baseline characteristics are presented in Table 1. Frequencies, means with standard deviations, or medians with inter-quartile ranges were calculated for the demographics and anthropometric measures depending on the distribution of the data. Nine children were normal weight at baseline. The normal weight children were included due to miscommunications between the municipality and a few of the school nurses performing the recruitment. Groups are similar at baseline in all aspects, with the exception of a larger number of children at risk of having movement difficulties in M_Ball_ in the SIA (10 vs. 3). The flow of participants is shown in Fig. 1. School nurses examined 3750 children (91.3 % of all 5th grade children during the two sampling years). Of these, 633 children were invited to participate in the OOIS and 115 children and their families accepted. Nine children withdrew before baseline measurements. Loss to follow-up after 52 weeks was 19 % and 32 % in the DCIA and SIA, respectively. Most participants who dropped out of the trial did not provide any reasons (72 %). For children randomized to the DCIA being of non-Danish ethnicity, there was an increased risk of missing more than one measurement occasion (*P* = 0.038). In short the DCIA were effective in reducing children’s BMI and BMI z-score across 52 weeks compared to children from the SIA (manuscript in review).

**Table 1:** Baseline characteristics

	Total	Day camp intervention arm	Standard intervention arm
Total N (male %)	106 (44.3)	55 (47.3)	51 (41.2)
Age (years)	(*n* = 106)	*(n* = 55)	(*n* = 51)
Mean (SD)	12.0 (0.4)	12.0 (0.4)	12.0 (0.4)
Ethnic Danish (%), (% males)	66.0‡ (41.2 %)	70.6 (44.4 %)	61.8 (33.3 %)
SES ♂/♀ (N)^a^	(*n* = 99)	(*n* = 52)	(*n* = 47)
1	11/14	7/8	4/6
2	18/21	11/13	7/8
3	12/23	6/7	6/16
Pubertal stage ♂/♀ (N)	(*n* = 106)	(*n* = 55)	(*n* = 51)
1	4/0	3/0	1/0
2	24/4	13/2	11/2
3	17/37	9/18	8/19
4	2/15	1/9	1/6
5	0/3	0/0	0/3
Body height (m)	(*n* = 106)	(*n* = 55)	(*n* = 51)
Mean (SD)	156.0 (6.1)	156.4 (6.6)	155.5 (5.7)
Body weight (kg)	(*n* = 106)	(*n* = 55)	(*n* = 51)
Median (IQR)	60.1 (53.9 – 65.5)	61.3 (55.4 – 66.2)	59.2 (52.4 – 62.9)
BMI (kg/m^2^)	(*n* = 106)	(*n* = 55)	(*n* = 51)
Median (IQR)	24.3 (22.6 – 26. 9)	24.8 (22.8 – 27.1)	23.9 (22.5 – 26.9)
Overweight category	(*n* = 106)	(*n* = 55)	(*n* = 51)
Normal weight (N), (% males)	9 (55.6 %)	3 (33.3 %)	6 (66.7 %)
Overweight (N), (% males)	67 (41.8 %)	36 (44.4 %)	31 (38.7 %)
Obese (N), (% males)	30 (46.7 %)	16 (56.3 %)	14 (35.7 %)
Motor skills (standard scores)	(*n* = 106)	(*n* = 55)	(*n* = 51)
Manual dexterity, Mean (SD)	24.5 (6.5)	25.0 (6.4)	24.0 (6.7)
Aiming and catching, Mean (SD)^b^	18.8 (4.3)	19.8 (3.9)	17.7 (4.4)
Balance, Mean (SD)	22.0 (6.7)	21.2 (6.2)	22.8 (7.2)
Overall, Mean (SD)	65.0 (12.1)	64.8 (12.4)	65.3 (11.9)
Hand strength (kg)	(*n* = 106)	(*n* = 55)	(*n* = 51)
Mean (SD)	24.2 (4.7)	24.2 (5.0)	24.2 (4.3)
Jump height (cm)	(*n* = 106)	(*n* = 55)	(*n* = 51)
Mean (SD)	28.9 (5.3)	28.6 (5.2)	29.2 (5.6)
Cardio respiratory fitness (ml O2/min/kg)	(*n* = 86)	(*n* = 80)	(*n* = 75)
Mean (SD)	34.0 (5.3)	33.2 (5.4)	34.8 (5.1)
Over physical fitness z-score	(*n* = 74)	(*n* = 55)	(*n* = 51)
Mean (SD)	−0.00 (77)	−0.04 (0.78)	0.04 (0.76)

Means with standard deviations for normal distributed data and, alternatively, medians with inter quartile ranges for skewed data are presented for each intervention arm and for the total sample

*SD* standard deviation. *IQR* Inter-quartile range. ^a^Based on the mothers’ education level. ^b^Significant difference between intervention groups. *BMI* body mass index. Motor skills are based on the Movement Assessment Battery for Children – second edition. *SES* Socio Economic Status

### Motor skills

As presented in Table 2, M_Overall_ remained unchanged between groups during the entire trial. M_Ball_ was improved in children from the SIA both after six (mean group difference; −2.2 (−4.1 to −0.4), *P* = 0.02) and 52 weeks (mean group difference; −2.7 (−4.6 to −0.8), *P* = 0.01) when compared to children from the DCIA. Compared to children from the SIA, children from the DCIA improved M_Balance_ (mean group difference; 3.5 (0.5 to 6.4), P = 0.02) after six weeks, but not after 52 weeks. M_Hand_ showed no change between groups at either follow up.

**Table 2:** The development of motor skills

	M-ABC-2 standard score Mean (SD)			Difference in change at 6 weeks		Difference in change at 52 weeks	
Variable	Baseline	6 weeks	52 weeks	Mean	*P*-value	Mean	*P*-value
	(*N* = 51/55)	(*N* = 43/51)	(*N* = 37/48)	(95 % CI)		(95 % CI)	
Manual dexterity							
Standard	25.0 (6.4)	26.0 (6.6)	27.4 (6.0)	−0.95 (−3.4 to 1.5)	0.45	−1.4 (−4.0 to 1.1)	0.27
Day Camp	24.0 (6.7)	24.4 (5.4)	25.6 (6.4)				
Aiming and catching							
Standard	17.7 (4.4)	20.3 (4.8)	20.3 (5.7)	−2.2 (−4.1 to −0.4)	0.02	−2.7 (−4.6 to −0.8)	0.01
Day Camp	19.8 (3.9)	20.3 (4.4)	19.4 (4.1)				
Balance skills							
Standard	22.8 (7.2)	23.6 (8.3)	26.8 (6.7)	3.5 (0.5 to 6.4)	0.02	−0.4 (−3.4 to 2.7)	0.81
Day Camp	21.2 (6.2)	25.7 (6.4)	25.2 (6.3)				
Overall motor skills							
Standard	65.3 (11.9)	69.9 (13.3)	74.5 (12.4)	0.3 (−4.7 to 5.3)	0.91	−4.6 (−9.7 to 0.6)	0.09
Day Camp	64.8 (12.4)	70.5 (11.2)	70.4 (12.4)				

The absolute values and the difference in change of the motor skill subscales and the overall motor skills from the Movement ABC-2 test standard scores

### Physical fitness

As presented in Table 3, F_Overall_ was higher in favour of the DCIA children at both follow-up measurements when compared to the SIA children (mean group difference; 0.28 (95 % CI 0.07 to 0.50, *P* = 0.01) and 0.24 (95 % CI 0.02 to 0.46, *P* = 0.03) at 6 and 52 weeks, respectively). F_Cardio_ was higher in children from the DCIA after 6 weeks (mL O_2_/(min · kg) mean group difference; 3.20 (95 % CI 1.36 to 5.04, *P* < 0.01), but no difference was observed after 52 weeks when compared to children from the SIA. No significant group differences were present at any follow-up with respect to F_Strength_ and F_Jump_.

**Table 3:** The development of physical fitness

	Physical fitness Mean (SD), N = SIA/DCIA			Difference in change at 6 weeks		Difference in change at 52 weeks	
Variable	Baseline	6 weeks	52 weeks	Mean (95 % CI)	*P*-value	Mean (95 % CI)	*P*-value
Handgrip (kg)	*N* = 51/55	*N* = 43/51	*N* = 38/48				
Standard	24.2 (4.3)	23.9 (4.4)	27.2 (4.3)	0.45 (−0.81 to 1.70)	0.49	0.96 (−0.35 to 2.26)	0.15
Day Camp	24.2 (5.0)	24.3 (4.5)	28.3 (5.8)				
Vertical jump (cm)	*N* = 51/55	*N* = 43/51	*N* = 38/48				
Standard	29.2 (5.6)	28.7 (5.6)	31.7 (5.5)	1.24 (−0.77 to 3.26)	0.23	1.27 (−1.51 to 4.05)	0.37
Day Camp	28.6 (5.2)	29.0 (4.4)	32.3 (5.9)				
Cardiorespiratory fitness (ml O2/min/kg)	*N* = 35/39	*N* = 33/39	*N* = 23/31				
Standard	35.1 (5.2)	35.4 (6.1)	36.6 (5.4)	3.20 (1.36 to 5.04)	<0.01	1.17 (−0.79 to 3.13)	0.24
Day Camp	33.5 (5.2)	37.2 (6.5)	37.3 (4.7)				
Overall physical fitness	*N* = 51/55	*N* = 43/51	*N* = 38/48				
Standard	0.04 (0.76)	−0.06 (0.79)	−0.11 (0.72)	0.28 (0.07 to 0.50)	0.01	0.24 (0.02 to 0.46)	0.03
Day Camp	−0.04 (0.78)	0.08 (0.68)	0.04 (0.80)				

The absolute values and the difference in change of fitness related components and the summed z-score of the components (physical fitness z-score)

## Discussion

When children’s MS and PF was compared across the intervention arms, the DCIA children improved their F_Overall_, but not the M_Overall_ when compared to the SIA. Changes in the motor skill subscales were ambiguous as M_balance_ was improved in children from the DCIA after six weeks, while children from the SIA improved their M_Ball_ after both six and 52 weeks. Improvements were not observed at any time in other subscales of motor skills and the F_Cardio_ after six weeks was the only sub-component improved in F_Overall_ for children from the DCIA compared to children from the SIA.

Our findings on DCIA improvement of M_balance_ after six weeks are in line with the ones in a study by D’Hondt and colleagues [13]. The authors investigated the development of gross motor co-ordination during a residential weight-loss intervention in overweight children with PA exposure comparable to the OOIS. When testing gross motor skills using the Körperkoordinationstest für Kinder (KTK), D’Hondt et al. found improvements in the overall KTK performance after 16 weeks. The KTK is relatively comparable to the M_Balance_ subscale of the M-ABC-2, where we in the OOIS also observed improvements in the DCIA. The length of stay for the participants at the camp was 16 weeks in the program evaluated by D’Hondt et al. compared to only six weeks in the OOIS. Nonetheless, post-camp balance improvements were observed in both cases, indicating that six weeks of intervention is sufficient in order to attain improvement in overweight children’s balance skills. If more sustained effects are desired, different intervention components, specific motor skill enhancement training, might be required as earlier observed in a school based sample [29]. In general, earlier findings have shown that improvements of MS and PF are more likely to occur in cases where interventions include elements of both motor skills and physical fitness. This may explain why we in the present study were unable to observe long-term changes of M_Overall_. In a previous study by Morano et al., improvements of MS and PF were observed in obese children after engaging in an eight months intervention programme containing combined PA and MS elements [12]. Similar conclusions were reached in a meta-analysis, including various types of intervention and different samples of children (primarily children with developmental coordination disorder), where included studies aimed at improving MS [30]. However, only two studies from the meta-analyses included children with overweight. Therefore, improving MS seem achievable when the training aims for it.

We consider the contribution of the standard intervention programme on the development of any MS or PF related outcomes to be imperceptible. Still, we observed improvement of the M_Ball_ in the SIA children compared to the DCIA after six weeks. No within-group changes were observed at either follow-up in the DCIA children or in the SIA children from six to 52 weeks with respect to the M_Ball_. Therefore, the SIA improvement from baseline to six week follow up was the only significant change present for this subscale. This pattern suggests that either the six two-hour sessions in the SIA and/or the regular school setting was providing enough impact to significantly improve the M_Ball_ or, alternatively, that the SIA participants systematically or coincidently underperformed at baseline with respect to this particular subscale. The latter is the most likely scenario, as a significantly lower M_Ball_ subscale score at baseline was present in the SIA compared to the DCIA (M_Ball_ mean difference; −2.1 (95 % CI −3.7 to −0.5, P = 0.01)), while no other MS or PF related outcomes differed between intervention arms at baseline. At six and 52 weeks follow up, the SIA children had an improved their score to be on the same absolute level as the DCIA children.

F_Overall_ was improved in children from the DCIA compared to children from the SIA at both follow-up measurements. Previous (non-randomized controlled trials) camp-based studies have reported similar short-term (post camp) findings [31–36]. However, none of these studies added measures of motor skills to supplement physical fitness and typically only included one or two physical fitness-related components (e.g. one-mile run). Why improvements were observed in the F_Overall_ in the day-camp children, while none were found in M_Overall_, could be a result of the day-camp intervention primarily used fitness enhancing elements with no specific motor skill training. Another plausible explanation could be that the response time on the development of motor skills is longer compared to physical fitness. Thus, both exposure time and intervention content could preferably be considered when aiming to improve both MS and PF during weight-loss programmes.

The improvement in F_Overall_ after 52 weeks was present despite any of the composing fitness outcomes showing any significant change at this time point. The directions of the PF sub-component effect sizes indicate that all three have contributed to the overall the day-camp improvement. This is further confirmed in the z-scores of the components (data not shown).

Post hoc regression analyses revealed that while improvements in BMI z-score were significantly associated with an improvement in F_Overall_ for all children combined, both after six and 52 weeks, no such association was present for M_Overall_. To some extent, this is in line with the main findings; improvement of physical fitness was associated with weight loss, while the association between M_Overall_ and weight loss was insignificant.

### Strengths and limitations

Strengths included the randomised design and relatively long follow-up period compared to earlier studies. The study is one of the first to investigate the development of both motor skills and physical fitness as a result of overweight children participating in an immersive weight-loss intervention program. Other important strengths of the study include free of charge participation in the OOIS, that the OOIS program already was implemented in the municipal system, and that screenings were part of an existing assessment in schools. This means that the program is more accessible for children and families who otherwise lack the resources to voluntarily sign up and pay for participation in weight-loss programs.

The M-ABC-2 battery showed ceiling effects in one-legged hopping and drawing trail (data not shown) and floor effects in backwards balance (data not shown). Taking into account the characteristics of the OOIS sample: overweight and relatively young children (baseline mean age 12.0) in the M-ABC-2 age band (11 to 16 yr), ideally they should not exhibit ceiling effects in any of the test items [37]. Ceiling effects are specifically unsuitable in intervention research, as parts of the improvement then cannot be registered. Another limitation concerning the M-ABC-2 test is the reporting of changes in motor skills merely through the product-oriented part of the Performance Test in the M-ABC-2 [6]. By including the process-oriented part of the M-ABC-2 (an assessment of movement quality during the test) or the Checklist (peer assessment of movement quality), the evaluation of motor skills could have been reported based on other dimensions as well. Consequently, despite recommendations of inclusion of both product- and process-oriented assessments [6], the motor skills development detected in the present study are restricted to the performance related part of motor skills.

## Conclusions

In general, the present study shows that only a small improvement in MS and PF can be attributed to participation in an immersive day-camp intervention focusing on weight loss through PA without specific MS training. For children in the DCIA, PF improved across one year follow-up, while MS remained unchanged when compared to children from the SIA. However, the day-camp participants did improve their balance skills after the intensive day-camp period relative to the standard intervention participants. Intervention programmes neglecting a focus on improving both MS and PF sub-components, are potentially restrained from providing the necessary skills for future PA participation that otherwise could favour the sustainability of an achieved weight loss. This could advantageously be considered in immersive intervention programmes, as they provide an otherwise optimal context for adding MS and PF improving content to the intervention.

## Abbreviations

BMI, body mass index; DCIA, Day-Camp Intervention Arm; F_Cardio_, cardiorespiratory fitness; F_Jump_, highest vertical jump height; F_Overall_, overall physical fitness; F_Strength_, handgrip strength; KTK, Körperkoordinationstest für Kinder; M-ABC-2, Movement Assessment Battery for Children – 2; M_Balance_, balance skills; M_Ball_, aiming and catching skills; M_Hand_, Manual Dexterity; M_Overall_, overall motor skills; MS, motor skills; OOIS, Odense Overweight Intervention Study; PA, physical activity; PF, physical fitness; SIA, Standard Intervention Arm; SES, Socio Economic Status; VO_2Peak_, maximum oxygen uptake
